# Echolalia from a transdiagnostic perspective

**DOI:** 10.1177/23969415221140464

**Published:** 2022-11-25

**Authors:** Tyler C McFayden, Shelia M Kennison, J Michael Bowers

**Affiliations:** University of North Carolina-Chapel Hill, Carrboro, NC, USA; 7618 Oklahoma State University, Stillwater, OK, USA; Virginia Tech, Blacksburg, VA, USA

**Keywords:** Echolalia, aphasia, autism, repetitive speech, transdiagnostic

## Abstract

**Background & aims:**

Echolalia, the repetition of one's or others’ utterances, is a behavior present in typical development, autism spectrum disorder, aphasias, Tourette's, and other clinical groups. Despite the broad range of conditions in which echolalia can occur, it is considered primarily through a disorder-specific lens, which limits a full understanding of the behavior.

**Method:**

Empirical and review papers on echolalia across disciplines and etiologies were considered for this narrative review. Literatures were condensed into three primary sections, including echolalia presentations, neural mechanisms, and treatment approaches.

**Main contribution:**

Echolalia, commonly observed in autism and other developmental conditions, is assessed, observed, and treated in a siloed fashion, which reduces our collective knowledge of this communication difference. Echolalia should be considered as a developmental, transdiagnostic, and communicative phenomenon. Echolalia is commonly considered as a communicative behavior, but little is known about its neural etiologies or efficacious treatments.

**Conclusions:**

This review is the first to synthesize echolalia from a transdiagnostic perspective, which allows for the direct comparisons across and within clinical groups to inform assessment, treatment, conceptualization, and research recommendations.

**Implications:**

Considering echolalia transdiagnostically highlights the lack of consensus on operationalization and measurement across and within disorders. Clinical and research future directions need to prioritize consistent definitions of echolalia, which can be used to derive accurate prevalence estimates. Echolalia should be considered as a communication strategy, used similarly across developmental and clinical groups, with recommended strategies of shaping to increase its effectiveness.

Echolalia is described as “parroting,” or reproducing previously heard sentences or words, not for the purposes of comprehension, clarification, or reframing (e.g., Cohn et al., 2022). Echolalia was first described in verbal and nonverbal (echopraxia) behaviors by Itard in 1825 (Itard, 1825; Roberts, 1989), refined by Pick (1924), and soon thereafter used by Kanner (1943) to describe the tendency of autistic^1^ children to repeat, as opposed to respond, when asked a question or provided with a verbal prompt. Throughout the years, researchers have varied in their definitions of echolalia, and the term echolalia has been applied to numerous presentations and clinical disorders, which remain largely siloed in their unique field of study. Echolalia has many linguistic functions ranging from communicative, compensatory, or self-stimulatory (Gernsbacher et al., 2016). Echolalia can be used as a way for individuals with limited self-generated language to communicate functionally (Gernsbacher et al., 2016); however, echolalia in some forms has been reported to negatively impact one's quality of life, contributing to lower social acceptance and reduced vocational and independent living opportunities (Paul et al., 2005). Despite echolalia being recently reviewed in several diagnostic-specific outlets, including reviews of echolalia in aphasia (e.g., Ota et al., 2020; Torres-Prioris & Berthier, 2020) and autism (e.g., Cohn et al., 2022; Gernsbacher et al., 2016; Luyster et al., 2022), no research to date has reviewed echolalia from a transdiagnostic perspective. Critically, given the prevalence of echolalia in clinical groups and the number of publications in the past decade exploring echolalia, a literature review that consolidates the findings from a transdiagnostic perspective and provides transdiagnostic clinical recommendations is warranted.

Use of transdiagnostic models includes increasing attention to commonalities and differences across groups by prioritizing emotional, behavioral, neural, and cognitive domains of overlap between disorders (Craske, 2012). Potential advantages of a transdiagnostic approach to psychopathological phenomena include increased efficiency of training and dissemination of evidence-based practices, improved clinical fit for clinicians, and potential improvements in clinician and client satisfaction (Marchette & Weisz, 2017). In this narrative review, we adopt a transdiagnostic framework to understand the commonalities and key differences of echolalia within and across clinical and non-clinical groups through a neurobehavioral lens, as recommended by recent echolalia reviews (Luyster et al., 2022). Through this approach, we are better able to inform empirically supported treatment recommendations, clinical assessment protocols, and highlight future areas for research. In this review, we integrate the available data on the presentations of echolalia including the prevalence, phenotype, and course throughout select disorders, including autism and aphasias. Second, we review neural hypotheses that have been proposed to account for echolalia. The neural section also discusses the brain regions known to be involved in producing echolalia. In the third section, we examine treatment approaches for echolalia, including behavioral and pharmacological approaches. Lastly, we provide future recommendations spanning the treatment, assessment, and research of echolalia in clinical settings.

## Method

The current review sought to evaluate a body of literature under an existing transdiagnostic theory and is thus considered a narrative review (Ferrari, 2015). A narrative review approach was chosen due to its ability to (a) include previously published review papers and meta-analysis on similar topics, (b) avoid quantitative bias from single studies, (c) generate ideas for future research, and (d) generate speculations for novel intervention approaches (Ferrari, 2015). Search terms were derived from key concepts that were transformed into keywords to optimize obtaining related articles and eliminating irrelevant articles. Search terms for the current review included a Boolean combination of relevant terms: “echo* AND (autis* OR ASD OR aphas* OR tourette OR GTS OR schizo* OR clinical)”. Searches were conducted in *PubMed*, *PsychInfo*, and *Google Scholar*. Google Scholar searches afforded the potential to include non-published results, as in the case of student theses or dissertations. No date range filters were applied to allow for historic and current conceptualizations of echolalia; however, only results published in English were included. Papers of all types were included, such as case-studies, review papers, randomized control trials, and systematic reviews. Inclusion criteria included echolalia as a primary outcome or characteristic of the sample studied; exclusion criteria included results having to do with animal or cell models, papers related to brain surgery outcomes (e.g., in the case of aphasia), reviews of echolalia in historic literatures (e.g., from English or History domains), or papers simply mentioning echolalia as a symptom group but without further discussion of echolalia (e.g., in the case of autism). References of selected articles were hand-searched for other relevant articles, books, or publications.

## Presentations of echolalia

### Echolalia in non-clinical groups

Echolalia is a common developmental phenomenon that has been observed in non-clinical individuals during states of fatigue, inattentiveness, and altered consciousness (Ganos et al., 2012; Stengel, 1974). When considering echolalia as imitation (acknowledging that clinical definitions of echolalia involve more than just imitation), it is easy to see how and why echolalia occurs at a high frequency between one and four years of age. Imitation and repetition are important behaviors for social learning and are often paired with explicit teaching strategies in early childhood to facilitate learning (Caldwell & Millen, 2009). Imitation is also important for developing expressive language (Adank et al., 2013). In early childhood, imitation has been thought to serve several purposes, including refining speech perception and production, learning new vocabulary, learning grammar and syntax, learning social rules such as turn-taking, and even learning to differentiate the self from other (Bruscia, 1982). In early work, Bloom et al. (1976) suggested that all children demonstrate a large proportion of echolalic speech in early toddlerhood, which decreased in proportion as mastery of language increased. Echoed speech may also present in non-clinical preschoolers due to certain dysregulated mood states including confusion, frustration, or anxiety (Foster, 1998). Beyond toddlerhood, verbal imitations are used in adolescence and adulthood for a variety of purposes, including reciprocity, to demonstrate listening behavior, or to affirm/emphasize another's experience (Bruscia, 1982). Verbal repetition also emerges to take other forms, including mockery, insults, humor, or impersonation (Berthier et al., 2017). Bruscia (1982) argues that echolalia appears in early childhood and are not considered a clinical concern if the following conditions are met: (1) the frequency of responses are developmentally appropriate, (2) the responses are selective and serve a function, and (3) the function is beneficial to the person and appropriate to the context. As a natural extension of those rules, echolalic utterances and verbal repetition may be considered atypical or interfering when (1) the verbal repetitions are intense or unusually frequent, (2) when the echoes are nonfunctional, and (3), when it is detrimental to expressive or receptive speech, and/or inappropriate for the context.

### Echolalia in autism

In such cases of atypical or interfering echolalia presentations in early childhood, many clinicians view this behavior as a key indicator of autism spectrum disorder (i.e., autism; Ganos et al., 2012), a neurodevelopmental disorder that affects as many as 1 in 44 individuals (Maenner et al., 2021). The core diagnostic criteria for autism are (a) deficits in social and communication reciprocity, including deficits in verbal and nonverbal communication, and (b) the presence of restricted and repetitive behaviors, which includes echolalia and repetitive speech (APA, 2013). Echolalia in autism has been reported across language modalities (e.g., Deaf, autistic signers; Shield et al., 2017), and in various cultures and languages (e.g., Shyamala et al., 2007). Estimates suggest that 75% to 100% of autistic children produce repetitive speech; however, these estimates have been predominantly assessed with autistic youth with limited expressive language and may not reflect estimates across the spectrum of autism more broadly (Mayes et al., 2011; Roberts, 2014; Rutter et al., 1967). Although high estimates of echolalia have been reported in partially-speaking or minimally-verbal autistic youth (e.g., Paul et al., 1987, Rutter et al., 1967), recent research has failed to observe significant relationships between autism severity and echolalia (Gladfelter & vanZuiben, 2020; van Santen et al., 2013). Despite a clear clinical and research consensus regarding a high degree of echolalia among autistic children (Ganos et al., 2012; Roberts, 2014), there is no consensus regarding the prevalence rates of echolalia in autism (Berthier et al., 2017; van Santen et al., 2013). Lack of consensus estimates may be due to deficiency of objective measures, an absence of agreement on a concrete definition of echolalia (see Table 1), or the fact that the majority of prior research has been conducted with outdated diagnostic criteria (van Santen et al., 2013). Taken together, assessing the prevalence of echolalia across a wide range of presentations within autism—including verbal youth and individuals who use alternative/augmentative communication approaches—remains of high importance.

**Table 1.** Echolalia glossary across clinical groups.

Term	Clinical group	Description
Ambient Echolalia or Echoing Approval	Aphasia, GTS	Production of echoes of comments or questions not directed to the individual, but instead to other people, reflecting disinhibition
Automatic Echolalia	Autism, aphasia	Echoing direct comments addressed to the individual, or responses occur with all speakers, utterances, or in a completely random fashion
Delayed Echolalia	Autism	Echoed response may occur minutes, hours, days, or weeks after hearing the initial statement
Effortful Echolalia	Aphasia	Laborious combination of verbal repetition after hearing a phrase + self-initiated speech; individuals are aware of their echolalia. Echolalia may be produced with slurred speech and distorted prosody
Exact Echolalia or Pure Echolalia	Autism, aphasia	Echolalia that is repeated in the exact fashion as the imitated statement; a precise duplicate
Immediate Echolalia	Autism	Echoed response occurs immediately after hearing a statement
Meaningful Echolalia	Autism	Functional, used in a communicative context, or to provide an answer to a question
Mitigated Echolalia	Autism, aphasia	Shortened, abbreviated, or slightly altered echoes of the initial statement
Nonfunctional Echolalia	Autism	Echolalia appears random, does not appear to serve a purpose
Palilalia	Autism	Repeating one's own words in a whisper or quiet volume immediately following a typical-volume utterance
Selected Echolalia	Autism	Responses occur due to a certain pattern, either with selected speakers or specific utterances

*Note.* GTS = Gilles de la Tourette syndrome.

When observed in autism, echolalia is commonly divided into two categories: immediate and delayed echolalia (Hetzroni & Tannous, 2004; Foxx et al., 2004; Neely et al., 2016; Prizant & Duchan, 1981; Prizant & Rydell, 1984; Rydell & Mirenda, 1994). Immediate echolalia occurs within a few seconds of an initial vocalization, whereas delayed echolalia can take place anywhere from after two communicative turns to days later (Neely et al., 2016; Rydell & Mirenda, 1994). Research that separates echolalia into these two components suggests immediate echolalia may be more common in autism (Rydell & Mirenda, 1994), and is a larger focus of treatment programs compared to delayed echolalia (Neely et al., 2016), although this is not consistent within the literature (Hetzroni & Tannous, 2004). Immediate and delayed echolalia can further be classified by how exact they are in their repetition, whether they be exact repetitions (Prizant & Duchan, 1981) or slightly changed (i.e., mitigated; Roberts, 1989). In a recent theoretical review, Luyster and colleagues (2022) encourage consideration of immediate and delayed echolalia as forms of nongenerative speech and mitigated echolalia as transitional speech, both laying the groundwork for generative forms of speech, including idiosyncratic language (e.g., neologisms, idiosyncratic phrasing) and pedantic language commonly observed in autism. Other terms used to describe echolalia in autism are defined in Table 1.

Of note, discussion about echolalia as a vocal repetition phenomenon in autism also warrants discussion of an overlapping phenomenon—vocal stereotypy (Rapp & Vollmer, 2005). Stereotypies are defined as repetitive and involuntary movements or vocalizations that persist in the absence of social consequences and are maintained by nonsocial reinforcement (Lanovaz & Sladeczek, 2011; Rapp & Vollmer, 2005). As such, vocal stereotypy can include repeating previously heard words, akin to echolalia (Mancina et al., 2000), and other behaviors such as humming (Taylor et al., 2005), making instrument sounds (Falcomata et al., 2004), or grunting (Ahearn et al., 2003; Pruccoli et al., 2021). Although this conceptual overlap is largely unaddressed in the echolalia literature, researchers seem to separate vocal stereotypy from echolalia due to their differences in content being repeated and functions (Pruccoli et al., 2021). In other words, whereas echolalia is limited to the verbal repetition of speech for a communicative function, vocal stereotypy can include repetitions of nonwords and is considered non-socially motivated. For these reasons and from recent recommendations (Pruccoli et al., 2021), the following review does not include vocal stereotypy as an echolalic utterance, although future research may wish to further define and aggregate these terms.

### Echolalia in other clinical groups

Echolalia can also co-occur throughout development with other echophenomena, including echopraxia (repetition of actions), echomimia (repetition of facial expressions), and others (Ganos et al., 2012). In these instances, the combination of echophenomena may be conceptualized as a tic, as in the case of GTS, or commonly called Tourette's. Although vocal and motor tics are core features of the disorder (APA, 2013), echopraxia and echolalia have been historically acknowledged as core features (Lees et al., 1984; Robertson et al., 1988) and echolalia has been identified as one of the three core factors of GTS in a principal component factor analysis (Cavanna et al. 2011). Individuals with GTS typically echo both others’ words and their own, although echolalia is most commonly part of their tic repertoire (Finis et al., 2012). To summarize, the presence of echolalia in early childhood through adolescence is often first considered as an indicator of autism; however, when paired with other echophenomena, may reflect the emergence of GTS.

Echolalia is encountered in several other clinical and medical populations, including intellectual disabilities, neurodegenerative dementias (Ota et al., 2020; Torres-Prioris & Berthier, 2020), schizophrenia (Lee, 2004), post-epileptic states, Alzheimer's (Da Cruz, 2010), Fragile X syndrome (Paul et al., 1987), Catatonia (Haroche et al., 2020), and Rubinstein-Taybi Syndrome (Chung, 1998). Echolalia has also been investigated in unique medical cases, such as a case study of a 20-year-old male with a germinoma surrounding the bilateral ventriculus lateralis (Suzuki et al., 2012), the case of a 57-year-old female with neuropsychiatric systemic lupus erythematosus (Zapor et al., 2001) in adolescent-onset of encephalopathy (Sharawat & Panda, 2021) and encephalitis (Gurrera, 2019). In rare cases, echolalia has been reportedly induced by pharmacological treatments, such when topiramate, prescribed to control seizures in an older adult, resulted in significant speech impairments, including echolalia (Akil et al., 2014). The fact that echolalia is observed in so many clinical presentations and/or clinical case studies suggests that this behavioral presentation may not be so unique—in fact, there may be core underlying functions and neurobiologies that contribute to echolalia's emergence across phenotypes. Answers to questions about underlying neurobiologies have arisen from studying echolalia in another patient demographic with clear brain-behavior relationships: individuals with aphasias.

### Echolalia in aphasias

Unlike echolalia in childhood, which is commonly considered a developmental phenomenon, echolalia in aphasias result from brain damage that directly contributes to these verbal repetitions. Traditional descriptions of aphasia posit language disturbances are due to tissue damage involving different cortical areas, including deep grey nuclei and white matter connections (Torres-Prioris et al., 2019). Currently, aphasia is best understood as damage predominantly to the left perisylvian region, or the language area (Kim & Caplan, 2016). Lesions or damage can occur because of many causes, including stroke, dementia, head injury, brain tumors, or infections, and often result in speech and language impairments including difficulties with expressive language or receptive language. Aphasias can vary in their severity and etiology; major classifications include Broca's aphasia, Wernicke's aphasia, conduction aphasia, and many more. Although individuals with various aphasias have lost some language ability, the desire and intent to communicate remains, which often results in speech errors, such as verbal repetitions, otherwise known as echolalia (Torres-Prioris et al., 2019).

Echolalia is a typical accompanying feature of transcortical aphasias, which represent up to 20% of all aphasias (Berthier, 1999), and echolalia has also been described during the recovery process of classical perisylvian aphasias (global, Wernicke, conduction, Broca; Brown, 1975). Despite their high frequency in this diagnostic group, again there are no studies on the prevalence rates of echolalia in aphasic conditions (Berthier et al., 2017). Echolalia in aphasia has a heterogenous presentation and can take multiple forms, both between and within individuals, ranging from impulsive and non-communicative (i.e., ambient and automatic echolalia) to more voluntary repetition (i.e., mitigated echolalia; ME), with approval echolalia and effortful echolalia residing somewhere on the spectrum between the two extremes (Berthier et al., 2017). One of the most common types of echolalia in aphasia, ME, is theorized to be a compensatory strategy to improve verbal comprehension by verbally repeating previously heard utterances to integrate them into auditory working memory (Berthier et al., 2017). Moreover, ME can interfere with communication above and beyond aphasia alone, as it is a byproduct of deficits in auditory short-term memory, inhibition, and attentional control. Subsequently, echolalia often has negative consequences, including adverse impacts on attentional control and social withdrawal (Berthier et al., 2017; Torres-Prioris et al., 2019). Despite these deficits in memory and inhibition, ME can have some communicative advantages, such as repeating phrases to integrate content into auditory working memory. Indeed, echolalia broadly may have functional or communicative advantages, described more in the section below.

### Functions of echolalia

Echolalia's functions have been investigated for decades (e.g., Buium & Stuecher, 1974). A recent review suggested functions of echolalia have been investigated via multiple methodologies, including looking at surface structure of the repetition, relying on the communicative partner for determining function, evaluating the interaction in the context of the surrounding environment, or evaluating alterations of speech prosody (e.g., tone, volume, inflections, rhythm) as indices of function (Cohn et al., 2022). Although newer conceptualizations consider echolalia as occurring within a communicative context, this was not always how echolalia was conceptualized (Cohn et al., 2022). Early research suggested that presence of echolalia was “auto-erotic and auto-aggressive” (Buium & Stuecher, 1974, p. 353). During the behaviorism movement, echolalia was reclassified as a self-stimulatory behavior (Gernsbacher et al., 2016). Echolalia has also been described as a lack of inhibitory control, such as the case in GTS (Ganos et al., 2012), and can have some non-communicative functions (Cohn et al., 2022). However, recent evidence suggests that echolalia may have more of an adaptive function instead of being the result of a maladaptive neural function or deficit. In a factor analysis study using the Autism Diagnostic Interview-Revised (ADI-R), Lecavalier et al. (2006) showed that for autistic youth, echolalia did not load onto the factor associated with self-stimulatory, repetitive behaviors (e.g., hand flapping, repetitive requesting, repetitive use of objects, use of rituals). Instead, echolalia loaded onto a factor related to social communication, along with pronoun use and other linguistic strategies. In fact, autistic case studies have indicated that often, the primary maintenance factor for echolalia is social attention (Rehfeldt & Chambers, 2013). Considering echolalia as having communicative functions may provide further insight into its maintenance, etiology, and ways to shape or intervene, if necessary (Cohn et al., 2022).

Several providers and researchers maintain that echolalia is essential to the development of more productive language (Gernsbacher et al., 2016). Even in the early 1980s, researchers categorized echolalia as driven by linguistic functions, including declarative, turn-taking, self-regulatory, yes-answering, and requesting; over 30% of utterances were turn-taking in nature, and 27% were declarative vocalizations (Prizant & Duchan, 1981; Prizant & Rydell, 1984). Specifically, mitigated echolalia (ME) has garnered the most attention for being a productive way to expand one's vocabulary by slightly modifying previously heard utterances. For instance, some children repeat a sing-song phrase from a show while inserting their own words, which demonstrates the use of echolalia as a template for future language development. In fact, ME reportedly increases as expressive vocabulary increases, and then decreases as comprehension and receptive language begin to simultaneously increase, suggesting that ME is being used as a scaffolding strategy during a period of language growth (Roberts, 2014). When echolalia is observed with bodily cues, research suggests some autistic youth demonstrate physical and verbal imitation (e.g., bodily alignment when producing echolalia), which promotes co-orientation and can be used to initiative a conversational sequence (Kawashima & Maynard, 2019). The consensus from these findings suggests echolalia may not represent deficits such as behavioral maintenance or a lack of linguistic awareness but may instead represent a different approach to verbal communication, reciprocity, and social interaction.

### Summary

To summarize, echolalia has a variety of subtypes, depending on the psychopathology, which results in difficulties comparing clinical presentations given the lack of consensus definitions. However, diversity appears to be the rule, not the exception, as presentations of echolalia are as heterogeneous between clinical groups as within, which further underscores the clinical utility of approaching echolalia from a transdiagnostic perspective. Despite the heterogeneity of presentations, many similarities are present across and within disorders, including the presentation of multiple forms of echolalia within an individual, transitions between forms of echolalia during the course of the disorder or development, and the underlying communicative intent inherent in most echolalic utterances. The next section focuses primarily on neural mechanisms of echolalia; of which a good amount is gathered from research with aphasias. However, no works have applied the neural knowledge of aphasia to other clinical groups, which again opens a path for a transdiagnostic approach to glean more clinical information regarding treatment.

## Neural mechanisms

To date, the neural mechanisms underpinning echolalia across disorders are not fully understood. Numerous brain areas have been implicated in echolalia, including the right hemisphere, Perisylvian Language Area, Supplemental Motor Area, Anterior Cingulate Cortex, and the mirror neuron system. To best understand the intricacies of neural mechanisms involved in echolalia, we first provide a brief review of the neural underpinnings of speech and language broadly, followed by those related directly to echolalia.

**Figure 1.**
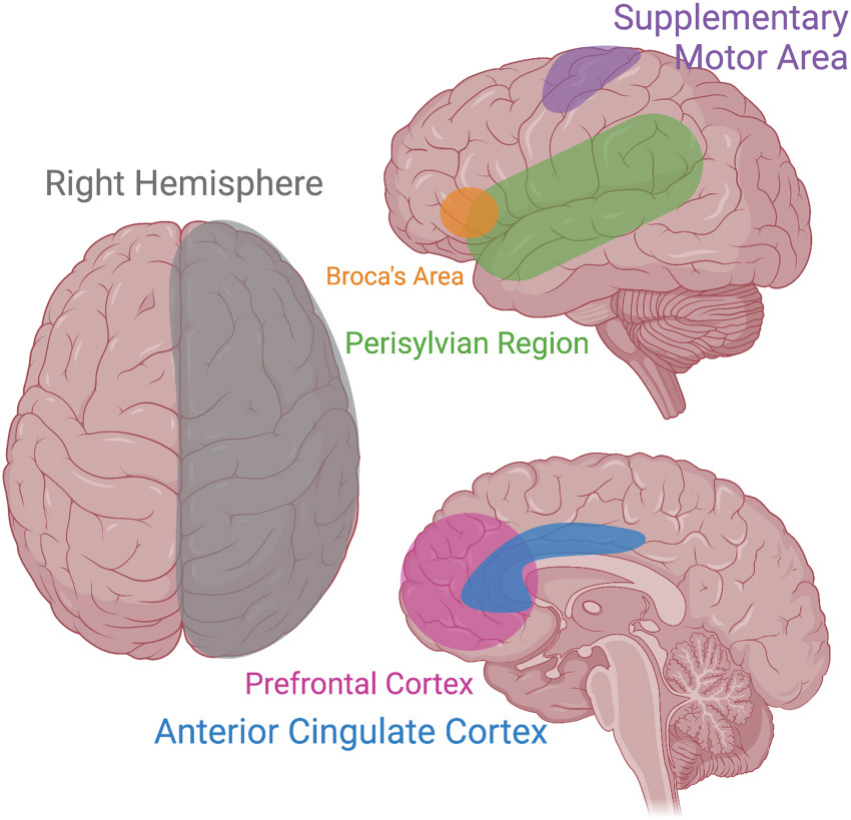
Several brain regions have been implicated in echolalia through the predominant study of aphasias. Areas of interest include the right hemisphere, Broca's Area, the Supplementary Motor Area, the Perisylvian region, the Prefrontal Cortex, and the Anterior Cingulate Cortex. Image created using Biorender.

There are multiple neural mechanisms for typical speech production and language. Figure 1 is a graphic of many involved areas of speech and language, including echolalia. Two of the most well-known brain areas include Broca's area in the inferior frontal gyrus, and Wernicke's area in the superior temporal gyrus (Friederici, 2011). Broca's area is associated with speech production and, in tandem with working memory, contributes to an individual's ability to use verbal expression and spoken words (Stinnett et al., 2020). Damage to Broca's area results in a unique type of aphasia (i.e., Broca's aphasia), which is characterized by an inability to speak fluently and speech filled with syntactical errors, despite a maintained ability to comprehend language (Nasios et al., 2019). Conversely, Wernicke's area is primarily involved in language comprehension (Javed et al., 2020). Damage to Wernicke's area results in receptive aphasia, (i.e., Wernicke's aphasia), which manifests as a loss of comprehension despite maintenance of verbal expressive skills (Nasios et al., 2019). Both Broca's and Wernicke's areas are left-lateralized processes, which reflects the overall lateralization of the word production network (Ries et al., 2017). Other areas implicated in language and speech production include the middle temporal gyrus, the primary motor cortex, the inferior parietal and angular gyrus, as well as the insular cortex and basal ganglia (Ardila et al., 2014; Friederici, 2011; Macoir et al., 2013). A complete overview of the language-related areas of the brain can be found in Price (2012).

### Right hemisphere theory

Despite numerous studies, clinical studies, and experimental studies demonstrating the importance of the left hemisphere for language production, many neuroimaging studies also demonstrate the important involvement of the right hemisphere in diverse language functions, including echolalia (Ries et al., 2017). Researchers have suggested that even in the cases of damage to the left hemisphere, the right hemisphere may contribute significantly to the development of echolalia in aphasias. Evidence supporting the *right hemisphere hypothesis* (Brown, 1975) includes case studies from aphasic patients and neuroimaging results from healthy adults. In aphasic clients, previously globally aphasic patients with large perisylvian language area lesions have developed echolalia several years after the onset of aphasia, which was hypothesized to occur via the gradual remodeling of the right hemisphere network (Berthier et al., 1997). In other case studies, individuals with aphasia developed mitigated echolalia due to residual activity in the left hemisphere dorsal stream and intact right hemisphere white matter tracts after extensive damage to the left hemisphere ventral stream (Berthier et al., 2018; Pulvermüller & Schönle, 1993). In healthy adults and in adults with transient virtual lesions in the left hemisphere inferior frontal gyrus, conducting a word repetition task yielded bilateral activation in the temporo-frontal areas, suggesting that when the left-brain areas are compromised, homologous brain regions in the right hemisphere or bilateral brain activation may contribute to verbal repetition demands (Hartwigsen et al., 2013).

Results from aphasias, therefore, suggest that recruitment of the right hemisphere may increase or exacerbate the presentation of echolalia. This unexpected lateralization (unexpected insofar as language is typically considered a left lateralized function) is also observed in autism (Escalante-Mead et al., 2003; Jouravlev et al., 2020; Lindell & Hudry, 2013). Developmentally, autistic youth may demonstrate a different developmental pattern of lateralization. Results from magnetoencephalography (MEG recordings) suggested that non-autistic youth start with bilaterally symmetric neuronal activation, which transitions to left lateralization with age. However, autistic youth transitioned from bilateral symmetric activity to rightward lateralization with age (Flagg et al., 2005). This reduced left lateralization has been directly related to language in minimally-verbal autistic youth (Nielsen et al., 2014), with a lack of left lateralization in connections involving Wernicke's area and the posterior cingulate cortex. Although the lack of left lateralization has not been directly linked to echolalia in autistic youth, Kleinhans et al. (2008) evaluated letter fluency and repetition in autistic youth. Results indicated greater activation in the right frontal and right superior temporal lobes for autistic compared to non-autistic controls, with the autistic group demonstrating overall reduced lateralization (suggesting over-recruitment of the right hemisphere). Collapsing findings across aphasias and autism, results suggest that an over-recruitment of the right hemisphere in language areas, specifically, may contribute to the emergence of echolalia.

### Perisylvian language area (PLA)

The left PLA has been implicated in auditory-visual short-term memory and verbal repetition skills of healthy adults (Koenigs et al., 2011). Accordingly, the PLA has been implicated in numerous types of echolalia seen in aphasia, including mitigated echolalia and automatic echolalia, where lesions are frequently observed in the left hemisphere outside the PLA (Berthier et al., 1997). Even in cases where the left hemisphere PLA remains intact after brain injury, PLA functioning can be disconnected from close cortical regions such as the Supplementary Motor Area (SMA) or the temporo-parietal cortex, which also underlie language production (Forkel et al., 2020; Hertrich et al., 2016). This isolation of the PLA has been referred to as the *isolation of the speech area* hypothesis, put forth by Goldstein (1948) and Geschwind and colleagues (1968).

### Supplementary motor area (SMA)

In addition to the LPA and right hemisphere/bilateral activation, the SMA has been implicated in schizophrenic and catatonic patients with echolalia (Haroche et al., 2020), in addition to patients with motor and vocal tics (Ganos et al., 2012). In patients with catatonia, where echolalia is omnipresent, researchers reviewed increased activation in the SMA compared to the excited form (which typically demonstrates more motor excitation such as stereotypy). The SMA has also been implicated in effortful echolalia in aphasia, which is currently understudied due to its lower prevalence rate compared to other forms of echolalia (Berthier et al., 2017; Hadano et al., 1998).

### Anterior cingulate cortex

Lastly, other brain regions including the anterior cingulate cortex (ACC) have been implicated in echolalia. In the case of a cerebral infarction which produced ambient echolalia, Suzuki et al. (2012) demonstrated damage to the left ACC and corpus callosum, which they indicated were responsible for the production of the ambient echoes. Ambient echolalia and echoing approval, broadly, tend to represent clear difficulties with disinhibition, as individuals with these types of echolalia will repeat words and phrases that were not directed to them, but spoken around them or to other people within earshot. In these types of echolalia, ACC has been implicated in addition to diffuse brain injury, extensive unilateral or bilateral lesions in the medial frontal and subcortical structures (Berthier et al., 2017).

### The mirror neuron system

Research suggests that the skills of repetition (repeating words) and imitation (repeating the phrase exactly, which includes prosodic features) are dissociable (Berthier et al., 2017). Structures affiliated with verbal imitation and repetition primarily include the audiovisual mirror neuron system located in the ventrolateral prefrontal cortex, superior temporal gyrus, and interior parietal lobule, all of which are under the supervision of an executive control network (Arbib, 2010). The executive control network is responsible for suppressing inappropriate or automatic repetition. In aphasias, where the brain pathology disrupts the regulatory function of these executive areas, verbal repetition occurs frequently and is not under explicit control, which promotes echolalia (Berthier et al., 2017). This pathway heavily implicates the audio-visual mirror neuron system, which has also been implicated in echolalia in Tourette's (Ganos et al., 2012), and in autism (see Williams et al., 2001, for a review). However, the mirror neuron system has also been criticized in numerous works given lack of consistent empirical support (e.g., Hickok, 2009) and its connection to echolalia warrants future research.

### Summary

Compiling results across studies and clinical populations, several areas of interest emerge (see Figure 1). Importantly, even in aphasia models where clear lesions have occurred, there are not yet clear causal mechanisms that contribute to echolalia—lesions do not cause reliable or predictable outcomes. These findings suggest that taking a lesion- or injury-specific approach to understanding echolalia may be missing a bigger picture. This provides an outstanding opportunity for a transdiagnostic process to be applied to the neural understandings of echolalia across clinical groups. This, in turn, provides a pathway to interventions improving speech and language outcomes for all individuals. A clearer understanding of echolalia as it relates to right hemisphere lateralization, or executive control mechanisms may expose future treatment options. For example, as executive function is a noted difference for many neurodevelopmental differences (Ozonoff, 1997), it is possible that targeting and treating behavioral inhibition broadly could also decrease the clinical interference of excessive frequency of echolalia as a by-product, while also improving executive function skills. Behavioral inhibition has yet to be the focus domain of current treatment approaches to echolalia; however, it warrants future research.

## Treatment approaches

When considering interventions for the presentation of echolalia, clinicians typically consider the entire individual on a case-by-case basis and focus on echolalia that is only considered “severe,” disruptive for expressive and receptive communication, and/or impacting quality of life (Berthier et al., 2017). As echolalia has been predominantly conceptualized as having communicative intent and function, echolalia alone does not require treatment (Gernsbacher et al., 2016). However, in more severe presentations, or when echolalia causes functional impairment in communication, social, or academic outcomes, treatments targeting the reduction of frequency and interference of echolalia may be suggested by clinicians, educators, or parents (e.g., Berthier et al., 2017). The current section reviews behavioral interventions for children and adults, followed by pharmacological interventions, and concludes with a summary and discussion of potential harms of treatment from a neurodiversity perspective.

### Behavioral interventions

A systematic review compiled and compared effect sizes for a variety of treatment programs for echolalia with autistic youth (Neely et al., 2016). Results indicated that no single intervention met criteria for a well-established or “evidence-based” approach to reducing echolalia. However, six unique studies and four overall therapeutic approaches were identified as “conclusive”: cue-pause-point, differential reinforcement, positive reinforcement of appropriate responses, and script training/visual cues (Table 2). Cue-pause-point language training is described as replacing echolalia with functional language labeling (Foxx et al., 2004). Foxx and colleagues demonstrated successful intervention by instructing children to remain quiet before and during a question and then to respond based on the cued presentation of images that indicated appropriate responses. In these differential and positive reinforcement paradigms, responses that are non-echolalic are taught and reinforced, while echolalic responses are ignored, punished, or re-shaped (Foxx et al., 2004). Script training plus visual cues use visual and behavioral scripts of appropriate social responses (i.e., visual cues when to stop talking by a picture of a face schematic with a finger over its lips) and has demonstrated clear reductions in echolalia (Ganz et al., 2008). Lastly, there are a few newer interventions that do not yet have conclusive evidence, including whole body vibration (Bressel et al., 2011), response interruption and redirection (RIRD, Pastrana et al., 2013), a computer-based intervention program (Hetzroni & Tannous, 2004), and a matched stimulation and RIRD (Love et al., 2012). Each of the aforementioned approaches reported immediate reductions in echolalia following intervention for children, with the longest follow-up indicating treatment effects persisted through 7 weeks (Li et al., 2022); however, no research has evaluated the duration of these treatment effects or the impact of echolalia reduction on quality of life for autistic youth. Furthermore, only one treatment modality was conducted with autistic females; the majority of intervention work has been conducted with autistic males only. Thus, these treatments warrant future research.

**Table 2.** Treatments for echolalia with conclusive evidence in autism spectrum disorder.

Intervention	Certainty of Evidence	Research Group(s)	Participants	Type of Echolalia
Cue-pause-point	Conclusive	Foxx et al. (2004); Foxx & Faw (1990); McMorrow & Foxx (1986); Valentino et al. (2012)	5- to 6-year-old males; 21-year-old adult males; 3-year-old male	Immediate
Script training and visual cues	Conclusive	Ganz et al. (2008)	7- to 12-year-old males	Delayed
Differential reinforcement of lower rates	Conclusive	Handen et al. (1984)	16-year-old male	Delayed
Tact modeling plus positive reinforcement of appropriate responses	Conclusive	Karmali et al. (2005)	3- to 4-year-old males and females	Delayed

*Note.* Adapted from Neely et al. (2016).

In studies involving adults, again researchers emphasize first asking and answering the question—does this presentation of echolalia warrant treatment (Berthier et al., 2017)? Research suggests that with aphasic adults, automatic echolalia in non-fluent transcortical aphasias may be the only available verbal production, and thus warrants redirection and sculpting into meaningful speech production. Treatment modalities aiming to teach or employ action-perception links, such as Constraint-Induced Aphasia Therapy (CIAT) has been demonstrated as effective in treating Wernicke's aphasia in combination with pharmacologic intervention (Berthier et al., 2018). CIAT is a behavioral, intensive treatment where the individual is forced to rely only on verbal communication in mass practice (Pulvermüller & Schönle, 1993). After isolating the action-observation and imitation network, two new models of behavioral interventions for aphasia were developed including computerized, project IMITATE (Intensive Mouth Imitation and Talking for Aphasia Therapeutic Effects; Lee et al., 2010; Sarasso et al., 2014) and Speech Entrainment (Fridriksson et al., 2012). Both treatments aim to improve aphasic speech via action observation and verbal feedback, ideally recruiting both the dorsal and ventral areas in both hemispheres. Although these treatment approaches have been used for speech recovery in aphasic adults, these interventions did not specify if these adults had echolalia. As such, the efficacy of these two action-imitation treatments for echolalia remain undetermined.

### Pharmacological interventions

Pharmacological intervention is not the first line of treatment for any presentation of echolalia. In select etiologies, such as aphasias, it is suggested that echolalic speech is a result of neural pathway dysfunction or neurotransmitter dysregulation and thus is best treated via pharmacological intervention. Most pharmacological interventions have been attempted in patients with stroke, aphasia (Bae et al., 2019), schizophrenia (Lee, 2004), and epilepsy (Cho et al., 2009). In one case study of an autistic adult, beta blockers (non-selective β1 and β2 antagonists propranolol and nadolol) significantly reduced symptoms of echolalia and stuttering during hospitalization (Schelke, 2017). As beta-blockers target receptors for catecholamines (e.g., norepinephrine) and because dopamine is a biosynthetic precursor of norepinephrine, there could be a future pharmacological benefit from targeting the family to reduce atypical sensory excitation in autistic youth (Joh & Hwang, 1987; Ludlow & Wilkins, 2016).

### Summary

Across all treatment approaches, similar themes of behavioral strategies emerged; pharmacological interventions appear to be the last resort. Behavioral strategies implicated in treatment of echolalia include clear instructions, behavioral modeling, positive reinforcement, script training, response interruption, and planned ignoring. Although no behavioral approach has been deemed “evidence-based” for autism (Neely et al., 2016), many traditional behavioral paradigms have demonstrated conclusive evidence for reducing the frequency, intensity, and impairment of echolalia- at least in the short-term. The lack of evidence-based approaches may reflect the nature of the treatment research, as most studies conducted have been single-case designs, case-studies, or pilot data with small sample sizes. Importantly, the use of a single-case design is not a limitation in itself, as single-case designs can be conducted in a rigorous and systematic fashion; however, when case studies lack this type of rigor, they can be difficult to generalize. No echolalia treatment research has evaluated whether reductions in echolalia contributed to improvement in well-being measures or quality of life, a crucial omission of treatment research to date.

A discussion on behavioral treatment approaches for echolalia would not be complete without discussing the reported harms and potential for harm associated with poorly implemented behavioral analytic or applied behavioral approaches (Dawson & Fletcher-Watson, 2021). Behavioral intervention approaches rarely report on harms (Bottema-Beutel et al., 2020a, 2020b), which can cause significant distress for autistic children, adolescents, and adults (Kupferstein, 2018). Considering the value of neurodiverse communication further underscores the importance of determining at what point treatment is warranted, what approaches will be used, and how treatment success will be measured. As previous researchers have suggested that treatment may be warranted in “severe” presentations of echolalia, these decisions are almost entirely made by parents, professionals, or teachers (Berthier et al., 2017), especially in the context of autistic youth. Due to a dearth of reliable assessment or measurement tools to identify when echolalia may warrant treatment and/or to what extent echolalia is interfering with an individual's life, it remains unclear at what point treatment is “warranted,” and at what expense (Dawson & Fletcher-Watson, 2021).

## Discussion

The current review discusses the presentations of echolalia throughout typical development, autism, GTS, and aphasias with the core purpose of viewing echolalia from a transdiagnostic perspective to inform evidence-based assessment and treatment procedures. Importantly, this review is not a systematic review, and thus considered echolalia transdiagnostically from a narrative perspective. Conducting a systematic review, with heightened rigor, represents an area of future research. Acknowledging this limitation, we can still recommend next steps in the areas of clinical assessment, treatment, and research. Implications for clinicians and researchers are available in Box 1.

Box 1 Transdiagnostic Implications for Clinicians and Researchers

Transdiagnostic Implications for Clinicians and Researchers

- Echolalia prevalence estimates are lacking. Echolalia is considered a common phenomenon in typical development and other clinical groups, and yet no prevalence estimates exist for any group. Establishing prevalence estimates is of high clinical and research utility.
- We need consensus definitions and clear assessment protocols. To aid in transdiagnostic assessment and treatment of echolalia, we must create consensus definitions across clinical presentations based on established criteria such as (1) function, (2) time-duration, and (3) severity, and operationalize quantifiable methods of measuring echolalia to correlates with other linguistic variables and to establish for whom treatment is recommended.
- Treatment for echolalia may not be warranted or evidence-based. In agreement with the majority of the literature, echolalia is considered communicative in function across clinical groups. Previous echolalia extinction research has demonstrated no significant benefit for social communication or language, despite conclusive evidence for decreased echolalia in the short-term. Paired with recent evidence suggesting potential harms of behavioral modifications and a lack of understanding of for whom treatment is effective, echolalia as a “problem behavior” should not require or warrant treatment. When recommended, transdiagnostic perspectives should emphasize using well-established social communication and expressive language interventions to harness echolalia as a communicative strategy instead of having echolalia-specific treatment modalities.
- If treatment is warranted, behavioral inhibition may be a next-step treatment target for echolalia. Aphasic lesions do not reliably predict echolalia, which suggest that global processes—namely behavioral inhibition—may be a contributing factor. Behavioral inhibition may be an outstanding transdiagnostic treatment target, behaviorally or pharmacologically, that may serve to improve executive function and have implications for echolalia.

### Presentation and prevalence

Verbal repetitions and imitations are common and persistent through approximately the first three years of life, whereby these processes contribute to social skills, academic skills, speech and language learning, and social interaction (Bruscia, 1982). Typically, after three years, verbal repetition begins to drop-off naturally, as toddlers begin relying on other styles of communication and learning (Ganos et al., 2012). If verbal imitation and repetition persist non-functionally, these patterns of speech are coined echolalia, and are commonly considered criteria of autism, although they can be indicative of other clinical disorders (Ganos et al., 2012). The prevalence of echolalia is not current nor agreed upon, likely due to several issues including echolalia measurement, agreement about definitions of echolalia, the wide heterogeneity of presentations, and the ability of echolalia presentations to shift within clients. These summaries further underscore pleas for objective and quantifiable methods to measure and operationalize echolalia made by other researchers in the field (e.g., Torres-Prioris & Berthier, 2020).

This review highlighted a key point in understanding the presentations and prevalence of echolalia: the diverse definitions used for echolalia throughout the literature, including in autism, aphasia, and other medical conditions (Table 1). Several researchers indicated multiple types of echolalia (e.g., 14 specific functions of echolalia; Prizant & Rydell, 1984) which vary from construct to construct and from disorder to disorder. The many definitions underscore the importance of arriving at a consensus for definitions of echolalia across and within disciplines. Arriving at a consensus definition would support interdisciplinary medical and provider teams and would support research on the prevalence and interference of echolalia, which to date have not been reliably reported due to differences in measurement and definitions. Deciding upon consensus language and definitions is crucial when working with patient populations, families, and medical providers, and when informing treatment plans. Indeed, consensus operational definitions would support moving echolalia toward a transdiagnostic model of care.

Lastly, a large part of understanding how echolalia changes over time, within and between individuals, is being able to quantifiably measure the behavior. In most studies, echolalia is commonly evaluated using open-ended conversations, behavioral observations, or unstructured measures in small samples or short time-periods (Torres-Prioris & Berthier, 2020). In clinical assessments, echolalia can be scored in certain measures of autistic behaviors (e.g., the Autism Diagnostic Observation Schedule; Lord et al., 2012), but are frequently done so coarsely using clinician report (e.g., rating on a scale of 0, 1, 2). Being able to objectively analyze presentations of echolalia does reply upon consensus definitions; however, being able to quantitatively evaluate echolalia will allow for researchers and clinicians to study the relationships between echolalia and other linguistic and cognitive variables, in addition to measuring treatment improvement or progress over time.

### Neural mechanisms

Regarding the neural mechanisms of echolalia, many researchers agree that the left hemisphere is implicated, including potential brain regions such as the PLA, SMA, and ACC (Berthier et al., 2017). Additionally, although language is often viewed as a lateralized function, evidence suggests the right hemisphere could play an important compensatory function in echolalia throughout the studies of individuals with aphasia and exploratory lateralization studies with autism. Although the neural mechanisms of verbal repetition and imitation are well understood, the brain areas implicated in the onset and treatment of echolalia are not as well understood. These limitations are, in part, due to the only echolalia neuroimaging studies to date taking place in aphasia. Applying our preliminary knowledge of the implicated brain regions in aphasia to autism may highlight relevant brain areas to investigate in neuroimaging studies of echolalia in this population, and further may highlight lateralization and/or behavioral inhibition as important transdiagnostic language pathways.

The neural models of echolalia in aphasia are well documented due to the nature of aphasia and the direct brain-behavior relationships. However, echolalia in autism and other disorders is not well understood at the neural level. Given the extensive research being conducted currently in autism, it would be of interest to investigate neuroimaging research that has already been conducted to explore the relationships between echolalia and hemispheric activation, particularly in reference to the PLA, right hemisphere lateralization, and prefrontal cortices. If neuroimaging studies are not feasible in autism due to the difficulty eliciting echolalia in vivo, additional work could be conducted investigating inhibition and echolalia, given the aphasia model of inhibition contributing to difficulties suppressing echolalic utterances.

### Treatment

Regardless of its typical or clinical presentation, echolalia serves a communicative function. Although previously considered non-functional or non-communicative (Buium & Stuecher, 1974), echolalia is reported to be communicative in nature- informing others of decisions, pauses, confirming, conveyance of emotional states, solidifying phrases in verbal short-term memory, or as a response to a question (Berthier et al., 2017). Considering echolalia as a functional and communicative endeavor is an essential reframe, opposed to considering echolalia as an externalizing or “problem behavior” warranting behavioral or pharmacological treatment. When the focus of intervention research, the review of treatment for echolalia highlighted numerous behavioral treatment approaches. In Neely et al.'s (2016) systematic review for autism, the authors identified four behavioral treatment approaches that have sufficient evidence to demonstrate their conclusive treatment of echolalia (see Table 2), including cue-pause point training, script training + visual cues, differential reinforcement, and tact modeling + positive reinforcement. In adulthood, treatment approaches included constraint-inducted aphasia therapy (CIAT) and the potential for the application of intensive mouth imitation and talking for aphasia therapeutic effects (IMITATE). The majority of these behavioral interventions noted include the use of behaviorism principles, or applied behavior analysis, which have been reported to cause harm for autistic youth (Dawson & Fletcher-Watson, 2021; Kupferstein, 2018) and have not been systematically evaluated in terms of potential harmful effects (Bottema-Beutel et al., 2020a, 2020b; Rodgers et al., 2020). Beyond these concerns, there are limitations to the current evidence-based treatment approaches designed to treat echolalia, including the use of case studies or small groups and a lack of longitudinal studies. Future research should seek to operationalize at what point echolalia may warrant treatment and for which individuals. If treatment is warranted, behaviorism principles can be applied using a neurodiversity-affirming approach geared towards improved quality of life for children and adults.

## Conclusion

Echolalia, commonly considered within a disorder-specific discourse, should be considered a transdiagnostic, functional communication strategy. Present in typical and atypical development, echolalia is a heterogeneous phenomenon with a wide range of operationalizations and definitions. Utilizing a transdiagnostic approach in our review highlights the lack of consensus on definitions, assessment, and prevalence rates. When considering echolalia from a neurodiverse communication lens, echolalia may not warrant treatment, extinction, or reshaping, and behavioral applications should be considered in a cost-benefit analysis. However, for individuals who report interference or diminished quality of life due to echolalia (e.g., in adults with aphasia), burgeoning behavioral treatment modalities have garnered some support. Future directions include improving our clinical definitions and assessments of echolalia to better understand and inform treatment modalities and outcomes.

## Notes

1. Identity-first language is used throughout to be consistent with clinical and research recommendations (e.g., “autistic” instead of “individual with autism spectrum disorder”; Bottema-Beutel et al., 2021; Kenny et al., 2016). Furthermore, in adherence to Bottema-Beutel's recommendations on inclusive language, typically developing samples are referred to as “non-clinical” samples.
